# Resonance frequency versus fixed 0.1 Hz breathing in HRV biofeedback: a four-week randomized comparison

**DOI:** 10.1038/s41598-026-53333-6

**Published:** 2026-05-19

**Authors:** Sylwia Sumińska, Andrzej Rynkiewicz, Mikołaj Szulczewski

**Affiliations:** 1 Central Institute for Labour Protection - National Research Institute, Warsaw, Poland; 2 Faculty of Psychology, University of Warsaw, Warsaw, Poland; 3 Research Group Health Psychology, KU Leuven, Leuven, Belgium

**Keywords:** Slow-paced breathing, HRV biofeedback, Resonance frequency, Mental health, Resting HRV, Health care, Neuroscience, Physiology, Psychology, Psychology

## Abstract

Heart rate variability biofeedback (HRVB) is increasingly examined as an adjunctive method for reducing psychological symptoms. Standard protocols involve identifying each participant’s resonance frequency (RF); however, it remains unclear whether this individualized calibration offers advantages over training at a fixed breathing rate of 0.1 Hz. We conducted a randomized trial with three groups (RF, 0.1 Hz, Control) to compare the effectiveness of a four-week HRVB intervention using an individually determined RF versus a fixed breathing frequency of 0.1 Hz. Participants (*N* = 88) completed pre- and post-intervention assessments of perceived stress, anxiety, depressive symptoms (DASS-21), and resting HRV. Both HRVB groups showed significant reductions in stress, anxiety, and depressive symptoms relative to the control group, with no meaningful differences between them. Bayesian analysis provided anecdotal evidence favoring the null hypothesis of no difference between groups. The intervention did not produce significant changes in resting HRV. The present study does not permit firm conclusions regarding whether breathing at the RF confers additional benefits over breathing at a fixed rate of 0.1 Hz or whether the two approaches differ in effectiveness. Future studies aimed at detecting small differences between these approaches should include larger samples.

**Trial registration**: The study was retrospectively registered at isrctn.com (Identifier ISRCTN17808563 date of first registration 20/01/2026, https//doi.org/10.1186/ISRCTN17808563). The full trial protocol and statistical analysis plan can be accessed via the registry.

**Supplementary Information:**

The online version contains supplementary material available at10.1038/s41598-026-53333-6.

## Introduction

According to WHO^1^, as many as 970 million people worldwide suffer from mental health problems. High levels of stress that persist over a long period (chronic stress) are an important risk factor and involve etiological mechanisms contributing to the decline of mental and physical health^2,3^. Chronic stress contributes to an increased risk of the emergence of depressive disorders^4–6^, anxiety disorders^7,8^, or cardiovascular system dysfunctions^9^, among others. Given that conditions influenced by chronic stress are widespread and impose a substantial burden on both individuals and society, numerous efforts have been made to develop interventions aimed at reducing chronic stress and its impact on health.

One widely used and tested method for reducing chronic stress and improving both mental and physical health is controlled slow breathing^10^. Among interventions based on slow breathing, heart rate variability biofeedback (HRVB) is one of the most extensively studied^11^. HRVB involves training in slow breathing guided by real-time feedback on heart rate fluctuations. Its goal is to maximize respiration-related oscillations in heart rate (respiratory sinus arrhythmia, RSA) and to identify the breathing frequency that produces the largest increase in HRV, which is then used for regular self-practice. Previous research suggests that HRVB may serve as an effective complementary intervention for improving both mental and physical health outcomes^11,12^. HRVB has shown efficacy in reducing depressive symptoms^13–15^, anxiety^16–20^, and perceived stress^11,16,21^.

Although the precise mechanisms by which HRVB exerts its effects remain unclear, one widely proposed explanation highlights the role of enhanced cardiovascular oscillations during training^22^. Increases in RSA are typically observed as breathing slows, with the most pronounced effects occurring around six breaths per minute (6 bpm). At this frequency, RSA synchronizes with blood pressure oscillations driven by baroreflex-mediated homeostatic blood pressure regulation (Mayer waves), resulting in a resonance effect and a substantial amplification of cardiovascular oscillations^23,24^. This resonance is hypothesized to exert its beneficial effects by stimulating baroreflex function and modulating afferent cardiovascular signaling pathways involved in autonomic regulation^22,25^.

Because the breathing rate at which RSA is maximized – known as the resonance frequency (RF) – varies slightly between individuals and can change over time, the original HRVB protocol proposed determining each person’s RF individually^26^. This individually determined RF is then used to guide paced breathing practice between HRVB sessions. While breathing at one’s own RF is theoretically considered the optimal approach, a review by Lalanza et al.^27^ indicated that approximately 35% of studies on HRVB used a fixed breathing frequency for all participants and still reported beneficial effects^11^. This raises the question of whether optimizing breathing frequency for each individual yields better outcomes than using a fixed rate, such as the commonly used 0.1 Hz. This issue is particularly important because determining an individual’s RF is time-consuming, prone to error, and requires careful analysis of ECG or PPG signals. However, studies have rarely directly examined this question.

One single-session study suggested that breathing at an individual’s RF could produce more favorable emotional and physiological responses compared to breathing at a rate just one breath per minute above RF^28^. Yet, because the comparison involved a faster breathing rate rather than a fixed frequency near the average RF, it is difficult to determine whether the effects were due to resonance-specific mechanisms or simply the result of slower breathing. Another study compared a multi-session HRVB intervention (six sessions over three weeks with a three-month follow-up) at individual RF with abdominal breathing at a fixed rate of 0.1 Hz in individuals with borderline hypertension, and reported a larger decrease in systolic blood pressure (but not diastolic), as well as greater increases in baroreflex sensitivity and HRV indices in the HRVB than 0.1 Hz group^29^. Thus, while some preliminary findings suggest that determining the RF may be important, whether individual RF assessment provides advantages over fixed-frequency slow breathing — particularly for long-term therapeutic protocols — remains unresolved. As Shaffer and Meehan^30^ noted, this is “an elephant in the room” that needs to be addressed empirically.

Another unresolved question concerns whether HRVB can produce lasting increases in resting HRV, and whether breathing at an individual RF plays a specific role in such potential effects. Recent systematic reviews and meta-analyses on slow-paced breathing and HRVB interventions have not provided a consistent picture regarding their influence on resting HRV. Although several studies have shown that both slow-paced breathing and HRVB increase HRV during training, meta-analytic evidence for sustained changes in resting HRV is limited. Laborde et al.^31^ reported a small overall effect restricted to RMSSD, and other reviews have yielded similarly inconclusive findings^13,32,33^.

To address these questions, we examined whether a slow-breathing technique based on an individual RF demonstrates greater effectiveness than HRVB training with a fixed breathing frequency of 0.1 Hz (6 bpm) in reducing self-reported stress, anxiety, and depressive symptoms. To this end, we conducted four-week HRVB interventions and additionally assessed whether applying an individual RF influences resting HRV, given that HRVB aims to increase HRV during training and resting HRV is considered one of its potential therapeutic targets.

## Methods

### Participants

Participants reporting high stress levels during the past month were recruited between January and February 2022 through social media postings and by contacting the HR departments of various organizations in Poland. Eligibility for the study was determined using the Cohen Perceived Stress Scale (PSS-10), with inclusion limited to individuals scoring in the 7–10 sten range. Additional inclusion criteria assessed via the survey were: age 25–40, absence of chronic diseases (e.g. diabetes), psychiatric disorders (e.g. depressive disorders, anxiety disorders), neurological diseases (e.g. epilepsy), cardiovascular diseases (e.g. hypertension, heart failure, myocardial infarction, arrhythmia), respiratory diseases (e.g. asthma), and substance addiction (e.g. alcohol, cigarette). Additional exclusion criteria included taking medications affecting the central nervous or cardiovascular systems (e.g., sleeping pills, antidepressants, anti-anxiety pills, and medications used for cardiovascular diseases), participation in psychotherapy or regular engagement in breathing-related practices (e.g., yoga, meditation, breathing exercises), and in the case of women, being pregnant.

A G*Power^34^ a priori power calculation for repeated-measures ANOVA within-between interactions to detect a small effect size *f* = 0.20, power (1 − *β*) = 0.80, and an alpha value of 0.05 provided an estimated sample size of 66 participants. As reported in the meta-analysis by Lehrer et al.^11^, HRVB produced a medium effect size for reducing anxiety and depressive symptoms, whereas the effect size for stress reduction was small.

To compensate for potential participant dropouts and ensure sufficient statistical power, the planned sample size was increased to 88 participants. The mean age of participants was 30.86 years (*SD* = 5.11). The study was completed by 80 subjects (51 women). Participants who discontinued home training did not attend the final assessment and were therefore excluded from the final analysis. Adherence to home practice was monitored by reviewing responses from a questionnaire in which participants reported their affective state before and after each breathing exercise.

### Scheme of the study

**Fig. 1:**
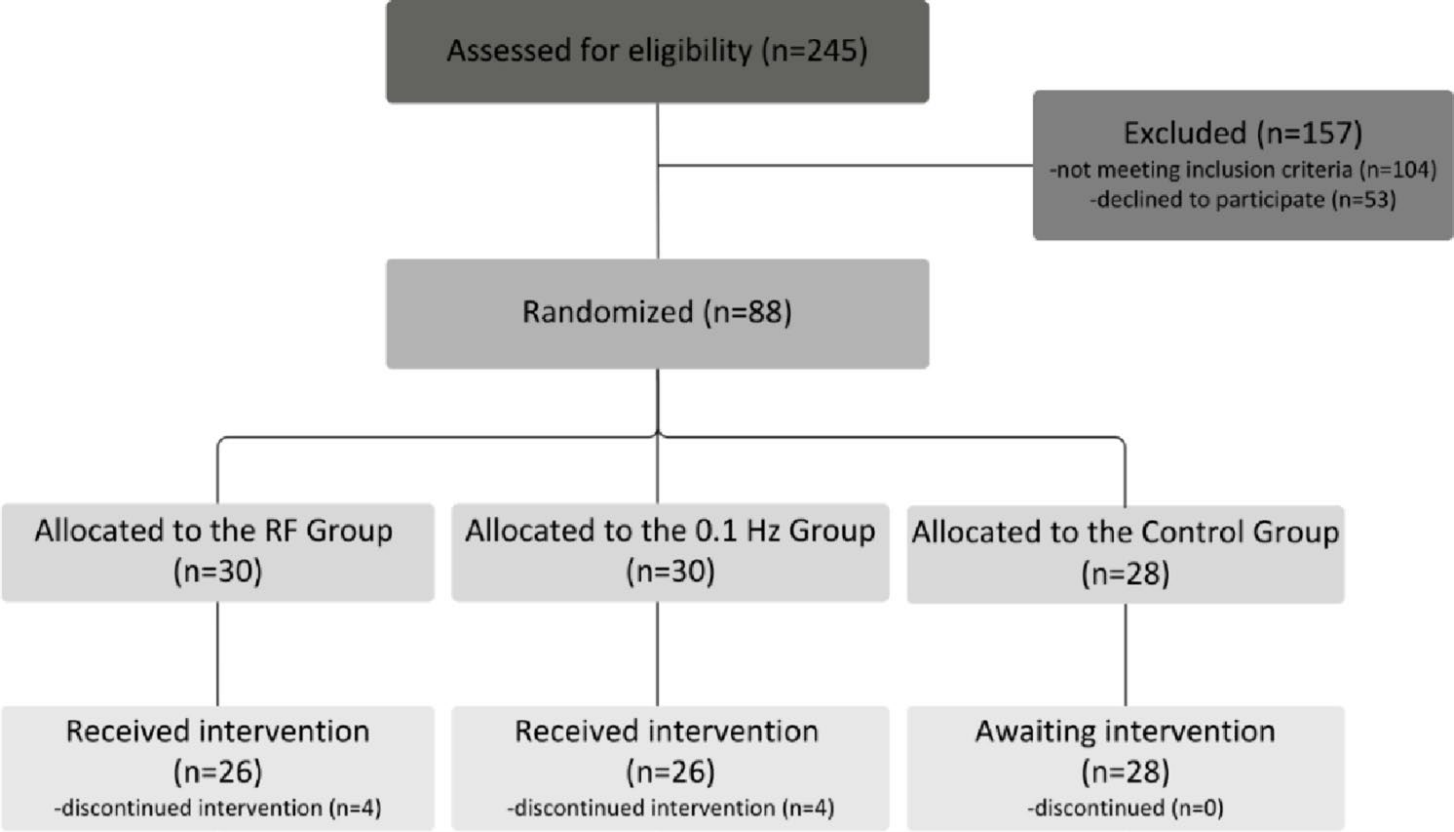
Study flow diagram: selection process of RCTs.

Participants were randomized to one of three groups using a computer-generated simple randomization sequence with a 1:1:1 allocation ratio. The study included three groups: (1) the RF Group, which practiced slow breathing based on HRVB with RF defined by a maximum LF HRV; (2) the 0.1 Hz Group, which practiced slow breathing based on HRVB with a fixed frequency of 0.1 Hz (6 bpm); and (3) a waitlist control group (see Fig. 1). Participants in both HRVB groups were unaware that the study tested the resonance frequency hypothesis, ensuring blinding to the specific intervention condition.

Qualified participants took part in an initial session during which self-reported levels of stress, anxiety, and depressive symptoms, as well as resting HRV, were assessed. Physiological responses and affective reactions to laboratory-induced stress and to paced breathing were also analyzed; however, these results are not reported in the present article.

**Fig. 2:**
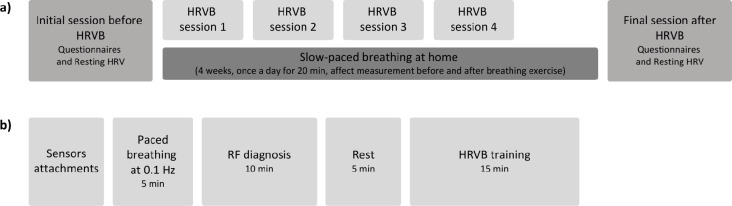
The scheme of the procedure: (**a**) the procedure of study, including initial and final laboratory sessions before and after HRVB, 4 sessions of HRVB, and 4 weeks of slow-paced breathing at home; (**b**) the procedure of HRVB sessions.

After completing the initial assessments, participants were assigned to one of three groups. Participants in the two intervention groups subsequently attended individual HRVB sessions once a week and practiced slow-paced breathing daily. The Control Group waited four weeks without training before attending the final session. After completing the final session, participants in the Control Group were offered the opportunity to participate in HRVB sessions. In the RF Group, the RF was determined at each weekly meeting, and participants then practiced slow breathing at the identified RF for the following week. In the 0.1 Hz Group, RF assessment was also conducted; however, the result did not affect the breathing rate, which was fixed at 0.1 Hz. Figure 2 illustrates the procedure for the four-week study and HRVB sessions.

The final assessment, conducted either at the end of the breathing training or after a four-week interval in the Control Group, followed the same procedure as the initial measurement. All measurements were taken at the same time of day for both sessions^35,36^. Participants were instructed to arrive well-rested, maintain their usual sleep routine the night before the session, refrain from heavy physical activity or alcohol consumption the day prior, and avoid consuming heavy meals, coffee, or energy drinks within two hours of the session^37^. To minimize potential bias from participants’ familiarity with the trainer during the stress induction task (not reported in this article), the study involved three experimenters. One experimenter conducted the initial and final assessments, while the other administered the HRVB training sessions. A third experimenter was responsible for group allocation. All experimenters involved in delivering the intervention received prior training in HRV biofeedback procedures.

The study was conducted according to the guidelines of the Declaration of Helsinki and approved by the Ethics Committee for Research with Human Participations of Warsaw University of Life Sciences (48/2021 dated 15.11.2021). Informed consent was obtained from all subjects involved in the study. Individuals in each group were paid PLN 650 (~€150) for participating in the study.

### Questionnaire tools

Cohen’s Perceived Stress Scale, PSS-10^38^. To measure the intensity of stress over the past month, we used a Polish version of the scale adapted by Juczyński and Ogińska-Bulik^39^. The scale was also used during the qualification for the study.

Depression Anxiety and Stress Scale, DASS-21^40^. The scale was used to measure the intensity of depressive and anxiety symptoms and the level of stress before and after participating in HRVB. It consists of three subscales, i.e. DASS-S (stress), DASS-D (depression), and DASS-A (anxiety), and refers to the intensity of symptoms over the past week. We used the Polish language version translated by Makara-Studzińska et al.^41^.

### Measurement of resting HRV

Resting HRV was measured using electrocardiography (ECG), with electrodes placed on the chest according to standard reference electrode placement procedures. HRV was analyzed using frequency-domain indices for the low-frequency (LF) and high-frequency (HF) bands, as well as the time-domain index, the root mean square of successive differences (RMSSD). LF and HF values were log10-transformed to correct for non-normal distributions. Frequency-domain HRV was calculated using the fast Fourier transform (FFT) method. Each periodogram was computed with a Hamming window applied to sequences of 256 samples extracted from the cardiotachogram at a sampling frequency of 2 Hz. Spectral power was then averaged within the 0.05–0.15 Hz (LF) and 0.15–0.4 Hz (HF) frequency bands.

Signals were recorded using a FlexComp Infiniti system and Biograph Infiniti software (Thought Technology Ltd., Montreal, Canada) at a sampling rate of 2048 Hz. Data were transferred to a computer via the FlexComp USB interface for further analysis.

### HRVB sessions and RF determination

The HRVB sessions followed the procedure described by Lehrer et al.^42^, with the total number of sessions reduced from five to four. Sessions included instruction in slow-paced diaphragmatic breathing, relaxation breathing, exhalation lengthening, guidance to avoid hyperventilation, and practice of RF or 0.1 Hz breathing using a visual pacer introduced in the first session. Each session began with a 5-minute slow-paced breathing period to introduce and practice these components; this period was extended during the first session to ensure correct technique. In subsequent sessions, instructions were briefly reviewed, and breathing technique was further refined. Participants then completed a 15-minute biofeedback training period during which RF or 0.1 Hz breathing was practiced with real-time feedback, allowing continuous monitoring and improvement of breathing performance. All instructions were delivered according to Lehrer’s protocol. Sessions were held individually once a week and lasted approximately 40 min. During HRVB sessions participants practiced slow-paced breathing while observing real-time feedback of heart rate (cardiotachogram) and respiration. HRVB training was conducted using Biograph Infiniti software and training materials provided by the equipment manufacturer (Thought Technology Ltd., Montreal, Canada).

The two HRVB training groups practiced slow-paced breathing at different frequencies. In the RF Group, the breathing rate corresponded to each participant’s individually determined RF, obtained during the RF assessment, whereas in the 0.1 Hz Group, it was fixed at 6 bpm for both HRVB sessions and home practice. The duration of inhalation and exhalation, both during sessions and home practice, was set at a 4:6 ratio, as recommended to enhance HRV and RSA^42,43^.

Participants were instructed to practice slow-paced breathing at home daily for 20 min. They were provided with a visual guide of their prescribed breathing frequency in the form of a screen-recorded video displaying a breathing pacer that maintained the 4:6 inhalation-to-exhalation ratio. The pacer included both visual cues guiding the breathing rhythm and auditory signals indicating the duration of inhalation and exhalation. Participants also received detailed instructions on how to perform the exercise correctly, in accordance with the procedures learned during the laboratory-based HRVB sessions. Physiological parameters were not monitored during home practice. Participants were instructed to discontinue practice if they experienced any symptoms of hyperventilation and to report any adverse effects to the study investigator. To monitor adherence, participants rated their affect before and after each self-practice session using an online questionnaire (detailed analyses of affect will be presented in a separate article). The structure of the home-based training is presented in Supplement 1.

The original HRVB training protocol describes the determination of RF during the initial training session, after which participants typically practice breathing at this frequency in subsequent sessions. The manual also indicates that RF may change over time and suggests adjusting the breathing rate if a different resonance pattern emerges during training^26^. However, it does not provide explicit guidance on whether RF should be reassessed systematically across sessions. Given empirical evidence suggesting that RF may vary between sessions^44^, we reassessed RF during each laboratory session in the present study to ensure that breathing practice was guided by the most current estimate of each participant’s resonance frequency.

Weekly RF assessment involved gradually decreasing the breathing rate in 0.5 bpm steps for 2-min intervals from 7.0 to 4.5 bpm, following the procedure described by Lehrer et al.^26^, with the upper limit of 7.0 bpm extended based on the recommendation of Capdevila et al.^44^. RF was defined as the respiratory rate producing the highest amplitude of HRV within the LF band, that is, the frequency with the largest absolute power^30,42^. Using this primary criterion allowed for standardization of the RF determination process. Additional indicators of increased RSA were also monitored, including phase synchrony between the respirometer and heart rate waveforms, peak-to-trough amplitude (HR Max – HR Min), and the maximum LF amplitude peak^30^. Supplement 2 presents absolute LF power values for each tested breathing frequency for all participants. HRV analyses were performed using Kubios software (University of Eastern Finland, Kuopio, Finland). Artifact correction included visual inspection of RR intervals, followed by an automatic correction algorithm with a very low threshold levels^45^. Table 1 presents the distribution of RF values across respiratory frequencies among study participants. During the assessment, the experimenter monitored physiological responses to ensure that participants performed the exercise correctly according to the breathing instructions.

**Table 1:** Number of participants (*N*) whose RF assessment indicated a resonance frequency at a given breathing rate (breaths per minute) across both trained breathing groups. HRVB = Heart rate variability biofeedback; RF = resonance frequency; bpm = breath per minute.

Week	Group	Results of weekly RF assessment					
		7.0 bpm	6.5 bpm	6.0 bpm	5.5 bpm	5.0 bpm	4.5 bpm
I	0.1 Hz Group	3	3	2	4	5	9
	RF Group	1	2	3	7	4	9
II	0.1 Hz Group	8	1	2	3	1	11
	RF Group	2	2	1	4	9	8
III	0.1 Hz Group	3	4	2	2	6	9
	RF Group	1	0	7	8	5	5
IV	0.1 Hz Group	2	3	5	2	6	8
	RF Group	3	1	5	3	5	9
Post-HRVB	0.1 Hz Group	3	1	3	3	8	8
	RF Group	4	1	0	5	8	8

### Statistical analysis

We used a frequentist approach complemented by Bayesian analysis. In the first stage, a two-factor repeated-measures ANOVA was conducted with the following design: session (initial, final) × group (the RF Group, the 0.1 Hz Group, the Control Group) to compare changes in subjective stress levels, anxiety, and depressive symptoms (DASS-21 scale) between groups. Statistical significance was assessed at the *α* = 0.05 level to determine whether the observed differences were statistically significant. As a post-hoc, independent-samples t-tests were conducted to determine whether groups differ in pre- and post-intervention changes in stress, anxiety, and depressive symptoms. The analysis was performed on change scores for the DASS-21 scale (post-pre) in both trained groups. Additionally, effect sizes were calculated for each group using Cohen’s *d*.

In the next step, a two-tailed Bayesian analysis was performed to evaluate the strength of evidence in favor of the null hypothesis (H₀) compared to the alternative hypothesis (H₁). The results were expressed as Bayes Factors (BF) and interpreted according to Jeffreys’ (1961) classification^46^. Combining both approaches provides a more comprehensive interpretation of the results – ANOVA indicates whether group differences are statistically significant, whereas Bayesian analysis assesses the extent to which the data support either H₀ or H₁. Bayesian analyses were also conducted using independent-samples t-tests on change scores of the DASS-21 scale (post-pre).

To examine whether resting HRV changed as a result of the HRVB intervention, a two-way repeated-measures ANOVA was conducted with the following design: session (initial, final) × group (the RF Group, the 0.1 Hz Group, the Control Group).

To explore whether the number of completed home self-practice breathing exercises predicted changes in perceived stress, anxiety, and depressive symptoms (DASS-21 scores), as well as changes in resting HRV, an exploratory linear regression analysis was conducted. In addition, a separate linear regression analysis was performed to examine whether changes in resting HRV predicted changes in stress, anxiety, and depression, as measured by the DASS-21.

Bonferroni correction was applied for post-hoc tests in ANOVA. The Greenhouse-Geisser correction was applied for data not meeting the assumption of sphericity. Analyses were performed in IBM SPSS Statistics 29 and JASP v. 0.19.3.0.

## Results

### Effect of four weeks of HRVB on mental health

We compared levels of stress, anxiety, and depressive symptoms before and after participation in HRVB in three groups. For the DASS-S (stress) dependent variable, ANOVA showed a main effect of the session (*F*(1,77) = 34.91; *p* < .001, *η*² = 0.312) and session x group interaction (*F*(2,77) = 8.506; *p* < .001, *η*² = 0.181). Post-hoc tests showed a significant reduction of stress level from initial to final session in the RF Group (*p* < .001) and in the 0.1 Hz Group (*p* < .001), but not in the Control Group. For the DASS-A (anxiety) dependent variable, a main effect of the session (*F*(1, 77) = 22.11; *p* < .001, *η*² = 0.223) and session x group interaction (*F*(2,77) = 5.5; *p* = .006, *η*² = 0.125) were observed. Post-hoc tests showed a significant reduction of anxiety symptoms in the RF Group (*p* < .001) and the 0.1 Hz Group (*p* = .002). For the DASS-D (depression) dependent variable, a main effect of the session (*F*(1,77) = 39.84; *p* < .001, *η*² = 0.341) and session x group interaction (*F*(2, 77) = 3.336; *p* = .041, *η*² = 0.08) were observed. Post-hoc analysis revealed a significant reduction of depressive symptoms in the RF Group (*p* < .001) and the 0.1 Hz Group (*p* < .001). Mean DASS-21 scores for the initial and final assessments in all groups are presented in Table 2.

An analysis of symptom severity based on established cutoff scores across all groups showed that a substantial proportion of participants (see Table 3) had scores in the clinical range, indicating clinically meaningful symptoms. These numbers decreased after the HRVB intervention in both trained groups. Effect size coefficients (Cohen’s d) calculated for post–pre differences showed slightly larger effects in the RF Group compared to the 0.1 Hz Group for stress (1.28 vs. 0.89), anxiety (0.83 vs. 0.59), and depression (1.04 vs. 0.99). However, these between-group differences did not reach statistical significance for changes in any of the symptoms, as indicated by independent-samples t-tests.

**Table 2:** Means (and standard deviations) of stress, anxiety, and depression levels at the initial and final sessions in all groups. DASS-S = stress; DASS-D = depression; DASS-A = anxiety (subscales of the DASS-21).

	DASS-S		DASS-A		DASS-D	
	Mean ± SD		Mean ± SD		Mean ± SD	
	Initial	Final	Initial	Final	Initial	Final
0.1 Hz Group	19.00 ± 8.97	12.38 ± 6.45	9.46 ± 6.13	4.77 ± 5.55	11.69 ± 7.43	6.54 ± 5.17
RF Group	20.62 ± 7.54	11.31 ± 6.14	11.31 ± 8.14	4.69 ± 5.48	14.62 ± 8.78	7.08 ± 7.09
Control Group	15.43 ± 8.02	15.07 ± 6.92	7.57 ± 5.03	7.36 ± 5.28	12.71 ± 9.85	10.21 ± 7.82

**Table 3:** Number of participants classified within the clinical range of DASS-21 (based on symptom severity) at pre- and post-HRVB assessment across all groups (the RF Group, *N* = 26; the 0.1 Hz Group, *N* = 26; the Control Group, *N* = 28). DASS-S = stress; DASS-D = depression; DASS-A = anxiety (subscales of the DASS-21).

	DASS-S			DASS-A			DASS-D		
	0.1 Hz	RF	Control	0.1 Hz	RF	Control	0.1 Hz	RF	Control
Pre	18	18	15	15	17	13	16	19	17
Post	8	5	14	5	7	13	7	7	14

While ANOVA indicates no significant between-groups differences in symptom change, this approach does not provide information about the relative probability of the null and alternative hypotheses given the observed data. To complement these findings and to evaluate the relative evidence for the competing hypotheses, a Bayesian independent samples t-test was conducted on the same data. The dependent variable reflected the change between post- and pre-intervention scores (post–pre), with more negative values indicating greater improvement. Since the analysis was non-directional, a two-tailed Bayesian independent samples t-test was performed, and the Bayes Factor (BF_01_). Bayes Factors (BF) were interpreted according to Jeffreys’ classification.

**Fig. 3:**
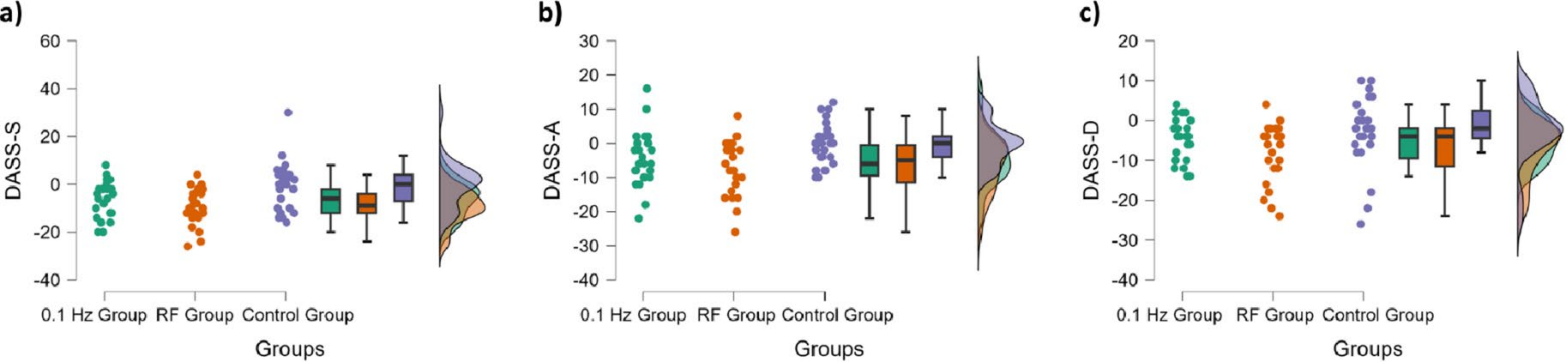
Raincloud plots for (**a**) changes in stress (DASS-S), (**b**) changes in anxiety (DASS-A), (**c**) changes in depression (DASS-D) in all groups (the 0.1 Hz Group, the RF Group, the Control Group).

Bayesian analysis results are shown in Table 4. According to Jeffreys’ classification, BF_01_ values indicate anecdotal, that is, inconclusive evidence slightly favoring the null hypothesis (H₀) over the alternative (H₁; see Table 4). Therefore, the findings provide weak evidence favoring the null hypothesis, indicating no clear differences between the RF Group and the 0.1 Hz Group in the change of stress, anxiety, or depressive symptoms (Fig. 3).

**Table 4:** Bayesian independent samples t-test comparing pre-post change scores (DASS-21) between the RF Group and the 0.1 Hz Group. BF_01_ = Bayes Factor in support of H0; DASS-S = stress; DASS-D = depression; DASS-A = anxiety.

Change of	BF₀₁	Error %
DASS-S	1.766	0.008
DASS-A	2.622	0.008
DASS-D	1.683	0.008

### Effect of four weeks of HRVB on resting HRV

**Fig. 4:**
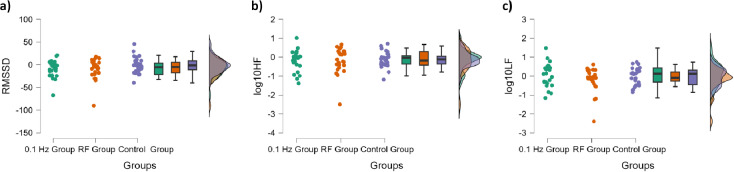
Raincloud plots for (**a**) changes (post-pre) in RMSSD, (**b**) changes in log10HF, (**c**) changes in log10LF in all groups (the 0.1 Hz Group, the RF Group, the Control Group).

The 2-factor analysis of variance revealed a significant main effect of the session for the resting RMSSD index, *F*(1,74) = 8.34, *p* = .005, *η*² = 0.101. The RMSSD was lower during the final session, regardless of group. Similarly, for the resting HRV-HF, a significant main effect of session was observed, *F*(1,76) = 6.25, *p* = .015, *η*² = 0.076, indicating lower HRV-HF during the final session across all groups. No significant effects were found for the HRV-LF. An additional analysis of differences in HRV change (post-pre), conducted using independent samples t-tests, showed no significant differences between either of the trained groups and the Control Group, nor between the two trained groups (Table 5, Fig. 4).

**Table 5:** Mean (and standard deviation) values of HRV indices (RMSSD, log10-transformed HF, log10-transformed LF) for the initial and final sessions in all groups.

	0.1 Group		RF Group		Control Group	
	Mean ± SD		Mean ± SD		Mean ± SD	
	Initial	Final	Initial	Final	Initial	Final
RMSSD	51.23 ± 25.94	42.24 ± 23.86	48.84 ± 30.07	41.04 ± 21.96	39.21 ± 17.11	37.47 ± 19.72
log10HF	2.87 ± 0.38	2.70 ± 0.50	2.87 ± 0.45	2.77 ± 0.44	2.72 ± 0.49	2.59 ± 0.53
log10LF	3.11 ± 0.44	3.18 ± 0.63	3.11 ± 0.47	3.01 ± 0.48	3.17 ± 0.49	3.14 ± 0.49

### Exploratory analysis of the relation between changes in well-being and adherence or changes in resting HRV

The mean number of completed home exercises was 21.44 (*SD* = 6.22; *Min* = 7; *Max* = 39). To examine whether adherence to home self-practice predicted changes in DASS-21 scores, an exploratory linear regression analysis was conducted. Results indicated that adherence did not significantly predict changes in DASS-21 scores (all *p* > .05). An additional regression analysis assessing whether changes in resting HRV between pre- and post-intervention predicted changes in mental health, as measured by the DASS-21, also revealed no significant effect (all *p* > .05).

## Discussion

This study examined the effects of HRVB training on self-reported stress, anxiety, and depressive symptoms, and tested whether breathing at an individual RF provides different outcomes compared with fixed 0.1 Hz breathing. To this end, we compared a group trained at an individually determined RF, as prescribed by the original HRVB protocol^26^, with a group breathing at a fixed frequency of 0.1 Hz, a common simplification in practice and research. Both HRVB groups showed reductions in self-reported stress, anxiety, and depressive symptoms after four weeks of training, whereas no improvements were observed in the Control Group. These results are consistent with previous studies reporting reductions in anxiety^16–18^, depressive symptoms^13,47^, and stress^11,16,21^.

The present study did not provide evidence for the superiority of individually determined RF over fixed 0.1 Hz breathing within a four-week HRVB protocol. At the same time, the data do not allow firm conclusions regarding whether the two approaches differ in effectiveness. These findings partly contrast with previous studies suggesting a stronger advantage of RF breathing. For example, Steffen et al.^28^ reported more favorable emotional and physiological responses during breathing at the individual RF compared with breathing one breath per minute above RF. However, these effects were observed during a single experimental session rather than following a longer-term HRVB intervention. Lin et al.^29^ compared RF breathing with fixed 0.1 Hz breathing and reported greater reductions in systolic blood pressure and larger increases in HRV in the RF condition, but this study was conducted in individuals with borderline hypertension, which may limit generalization to generally healthy but stressed populations.

HRVB increases HRV during training through biofeedback and produces a pronounced acute rise in HRV. It has been hypothesized that HRVB may also lead to sustained increases in resting HRV, indicating enhanced parasympathetic cardiac control and potentially representing a mechanism underlying its beneficial effects. Some studies have reported increases in resting HRV following HRVB^47–50^, whereas others have not observed such effects^51,52^. In the present study, resting HRV did not increase following HRVB. Instead, HRV (RMSSD and HF) decreased at the final assessment across all groups, including the Control Group, reflecting a main effect of the session rather than an intervention-specific effect.

The absence of significant increases in resting HRV is consistent with the broader literature, which remains inconclusive regarding the effects of HRVB on baseline HRV. Meta-analyses have reported mixed findings. Li et al.^53^ did not find evidence for an overall effect of HRVB on HRV, whereas Gathright et al.^54^ and Laborde et al.^55^ (including slow-breathing interventions beyond HRVB) reported small overall effects (*d* = 0.31 and *d* = 0.32, respectively). However, results across studies remain highly heterogeneous, with effects reported for different HRV indices. This heterogeneity likely reflects methodological differences, including limited clarity regarding HRV measurement procedures, selective reporting of HRV parameters, lack of control over respiratory rate, which may decrease during the intervention, and variation in intervention protocols. Several studies reporting positive effects were conducted in clinical populations, including individuals with depression^47–49^, whose baseline HRV may differ from that of healthy populations. In contrast, the present study involved generally healthy but stressed individuals whose baseline HRV may have been relatively high, leaving less room for improvement following the intervention.

Another explanation for the inconsistent effects of HRVB on resting HRV may be the duration of the intervention. In this study the training lasted four weeks, slightly shorter than the median duration of HRVB protocols (five weeks) reported in the meta-analysis by Lehrer et al.^11^. Although meta-analyses suggest that intervention length does not moderate overall HRVB outcomes^11,16^, they did not specifically examine resting HRV. Longer training periods may therefore be required to produce stable changes in resting HRV. Given the limitations of short-term HRV recordings and potential situational influences, future studies could use longer measurement periods, such as 24-hour HRV monitoring, to better capture the effects of HRVB on HRV.

Resting HRV has been associated with mental health, with lower levels reported in anxiety^56–58^ and depressive disorders^59–62^, although effect sizes are typically small. Some studies also suggest that symptom reductions following HRVB may be accompanied by increases in HRV^49,50^. To examine whether improvements in DASS-21 scores were accompanied by changes in resting HRV, we conducted an exploratory analysis. No such relationship was observed. These findings therefore suggest that improvements in self-reported mental health are not necessarily reflected in resting HRV.

Our results demonstrated improvements in mental health in the two groups that received HRVB training. However, the absence of an active control condition and the inclusion of only a waitlist control group limit our ability to determine whether these effects were specific to HRVB or driven by nonspecific factors. Previous research has shown that placebo responses may be shaped by contextual influences, including positive expectations, perceived credibility of the intervention, and interpersonal interactions^63–65^. Such factors may be particularly relevant in interventions targeting self-reported psychological outcomes. It is therefore possible that participation in a well-known breathing-based training influenced participants through contextual mechanisms that may account for a substantial proportion of the observed effects.

Another limitation of the present study is the absence of a follow-up assessment, which would have allowed evaluation of the durability of the observed effects. Incorporating longitudinal designs in future research could provide a more comprehensive understanding of the long-term impact of HRVB on mental health. In addition, home-based practice was not objectively monitored using physiological or device-based measures. Instead, adherence was assessed through an online procedure in which participants performed the breathing exercise using a paced instructional video and reported their affective state before and after each session. Although participants received detailed instructions and were asked to follow the same procedures as during laboratory sessions, the lack of objective verification represents a potential source of uncertainty regarding adherence to the prescribed training and the accuracy of its implementation.

In summary, our findings suggest that HRVB, both with individually determined RF and with a fixed breathing rate of 0.1 Hz, is associated with improvements in self-reported stress, anxiety, and depressive symptoms. However, the present study did not demonstrate that these improvements were accompanied by significant changes in baseline HRV after four weeks of training. Moreover, no evidence was found to support the superiority of individually determined resonance frequency over fixed 0.1 Hz breathing within the four-week HRVB protocol. Future studies should consider larger samples to increase statistical power and facilitate detection of potentially small effects, and examine these differences in clinical populations, where effects may be more pronounced due to lower baseline HRV and greater potential for improvement.

## Supplementary Information

Below is the link to the electronic supplementary material.

Supplementary Material 1

Supplementary Material 2

## Data Availability

The datasets generated and analysed during the current study are available in the Zenodo repository, https://doi.org/10.5281/zenodo.17733890.
