# Supplementary material for: Resonance frequency versus fixed 0.1 Hz breathing in HRV biofeedback: a four-week randomized comparison
Source: Sci Rep. 2026 May 19;16:22630. doi: 10.1038/s41598-026-53333-6 (PMC13381940; doi:10.1038/s41598-026-53333-6)
Supplement: Supplementary file 1 — Supplementary Material 1 [file 41598_2026_53333_MOESM1_ESM.docx]

**Supplement 2. Resonance frequency determination**

**Table 1. Absolute power in the LF band [ms²] at all breathing frequencies during five sessions in both groups.**

| Group 0.1 HZ | | | | | | | | Group RF | | | | | | | |
| --- | --- | --- | --- | --- | --- | --- | --- | --- | --- | --- | --- | --- | --- | --- | --- |
| No | **Sess.** | **Absolute power in the LF band [ms²]** | | | | | | **No** | **Sess.** | **Absolute power in the LF band [ms²]** | | | | | |
|  |  | **7.0 bpm** | **6.5 bpm** | **6.0 bpm** | **5.5 bpm** | **5.0 bpm** | **4.5 bpm** |  |  | **7.0 bpm** | **6.5 bpm** | **6.0 bpm** | **5.5 bpm** | **5.0 bpm** | **4.5 bpm** |
| 1 | I | . | 15250 | 14225 | 14324 | **15954^AB^** | 14811 | 1 | I | 12768 | **13418^ABC^** | 12530 | 9993 | 8061 | 4455 |
|  | II | 9856 | 14132 | 8518 | 13620 | 14759 | **14907^A^** |  | II | 6065 | 6076 | 3204 | 4099 | 2326 | **8252^A^** |
|  | III | 4984 | 12219 | 10465 | 10937 | 14127 | **14426^ABC^** |  | III | 2023 | 2541 | **4389^AB^** | 2285 | 3943 | 2583 |
|  | IV | 10771 | 12261 | 12586 | 13290 | **14642^AB^** | 13027 |  | IV | 4102 | 2901 | **6623^AB^** | 4707 | 5795 | 1003 |
|  | V | 5739 | 6812 | 9293 | 9879 | **14442^ABC^** | 11543 |  | V | **4963^ABC^** | 2758 | 2473 | 1124 | 825 | 1073 |
| 2 | I | 1974 | 3418 | 1972 | **3874^ABC^** | 3108 | 3419 | 2 | I | 6034 | 4985 | 5415 | **6564^AC^** | 6437 | 3114 |
|  | II | 3563 | **3777^AC^** | 2956 | 3496 | 2344 | . |  | II | **5946^A^** | . | . | . | . | . |
|  | III | 2246 | 2322 | **5135^AB^** | 4221 | 3796 | 3175 |  | III | 5602 | 5768 | 4062 | **6475^AC^** | 6032 | 6237 |
|  | IV | 1906 | 2732 | **3523^AB^** | 2927 | 1244 | 1392 |  | IV | 2452 | 3444 | 5337 | 4568 | **5465^ABC^** | 3391 |
|  | V | 2425 | 3344 | 2521 | 2928 | 3729 | **5806^ABC^** |  | V | 3211 | 4239 | 3321 | **4756^ABC^** | 4044 | 4790 |
| 3 | I | 9206 | **16065^ABC^** | 15396 | 8550 | . | . | 3 | I | 4473 | 4895 | 4225 | 5411 | 6092 | **7578^ABC^** |
|  | II | **11187^ABC^** | 10761 | 8053 | 9945 | 6517 | 6372 |  | II | 2453 | 3812 | 3659 | 4308 | 4398 | **4879^ABC^** |
|  | III | . | . | . | . | . | **3001^AB^** |  | III | 3633 | 3697 | **6074^AB^** | 2979 | 4662 | 2854 |
|  | IV | 4200 | 5262 | **8984** | 5574 | 5246 | 6724 |  | IV | 3470 | 3401 | **5083^A^** | 5011 | 2734 | 4772 |
|  | V | 2816 | 3015 | 3879 | 4626 | **4858^ABC^** | 4820 |  | V | 4015 | 3855 | 4959 | 6726 | **7532^ABC^** | 5933 |
| 4 | I | 4015 | 3769 | 9731 | 4774 | 5061 | **6653^B^** | 4 | I | 1288 | 1955 | 1472 | 911 | **2147^AC^** | 1993 |
|  | II | 4369 | 5411 | 5729 | 7282 | 8839 | **11430^ABC^** |  | II | 938 | 1490 | 1608 | 1538 | 1962^ABC^ | **2059^ABC^** |
|  | III | 5073 | 6403 | 6355 | 7489 | **9062^ABC^** | 9054 |  | III | 721 | 1183 | 1626 | **1676^AB^** | 994 | 1352 |
|  | IV | 9414 | 9484 | 10522 | 10112 | 13107 | **16071^ABC^** |  | IV | **18193** | 17447 | 13821 | 15641 | 17660 | 16243 |
|  | V | 7415 | 6123 | 6694 | 7856 | 9247 | **10062^ABC^** |  | V | 1140 | 1258 | 1950^ABC^ | 1408 | **1985^ABC^** | 1619 |
| 5 | I | **16224^AB^** | 13926 | 14949 | 13999 | 14967 | 14732 | 5 | I | 3948 | 3105 | 3130 | 4340 | 6910 | **6962^BC^** |
|  | II | 9744 | 13235 | 17011 | 16197 | 17349 | **21451^ABC^** |  | II | **2297^A^** | 1768 | 1878 | 1481 | 934 | 1417 |
|  | III | 16003 | 15503 | 15385 | 16074 | 13654 | **20312^ABC^** |  | III | 5286 | 2326 | 5183 | 3328 | 5005 | **5307^BC^** |
|  | IV | **18193^B^** | 17447 | 13821 | 15641 | 17660 | 16243 |  | IV | 7526 | 8420 | **11034^AB^** | 7434 | 6740 | 8212 |
|  | V | 12934 | 15471 | 15187 | 16772 | 18967 | **20147^ABC^** |  | V | **10532^ABC^** | 6033 | 7236 | 7102 | 7174 | 7593 |
| 6 | I | 8340 | 11213 | 9117 | 8757 | 14838 | **19383^AC^** | 6 | I | 1166 | 1676 | 1266 | 4088 | **7314^A^** | 4778 |
|  | II | 1709 | 1214 | 2027 | 2548 | 3530 | **6574^ABC^** |  | II | 1362 | 3538 | **3627^ABC^** | 2680 | 2904 | 3301 |
|  | III | 2365 | **2467^C^** | 1836 | 2150 | 2115 | 1830 |  | III | 304 | 1014 | **2811^ABC^** | 2543 | 1616 | 1967 |
|  | IV | 2337 | 3917 | 5899 | **6038^AC^** | 5496 | 4748 |  | IV | 1515 | 1654 | 1740 | 1989 | 2252 | **2880^ABC^** |
|  | V | 659 | 958 | 1637 | 1867 | 944 | **4719^AC^** |  | V | 1730 | 3575 | 3154 | 3242 | **4400^ABC^** | 2735 |
| 7 | I | 3209 | 4526 | 1981 | **16237^ABC^** | 1042 | 6035 | 7 | I | **4718^AC^** | 2979 | 2030 | 1600 | 1506 | 2763 |
|  | II | 5112 | 4542 | 7128 | 9348 | 9319 | **11118^C^** |  | II | 2293 | 1967 | 2518 | 2368 | **2638^C^** | 1824 |
|  | III | 10727 | 7148 | 13268 | **15967^ABC^** | 12727 | 12930 |  | III | **1836^AC^** | 705 | 650 | 1076 | 72 | 1118 |
|  | IV | 8269 | 7065 | 9353 | 11489 | 10430 | **14206^AB^** |  | IV | **3189^AB^** | 1715 | 2367 | 1343 | 1017 | 1629 |
|  | V | 1215 | 2275 | 5729 | **9838^AC^** | 5952 | 8505 |  | V | **7674^A^** | 5826 | 4259 | 5778 | 2458 | 4081 |
| 8 | I | 12729 | 12395 | 10023 | 12666 | 11727 | **13279^ABC^** | 8 | I | 18346 | 15982 | 18662 | 19069 | 20753 | **23573^ABC^** |
|  | II | 1329 | 1319 | 1571 | 2248 | 2421 | **2961^ABC^** |  | II | 21389 | 18393 | 20394 | 23856 | 20962 | **29050^ABC^** |
|  | III | 472 | 487 | 642 | 1708 | 1959 | **2760^ABC^** |  | III | 18533 | 21155 | 19723 | 27135 | **29973^AC^** | 26167 |
|  | IV | 616 | 1043 | **3002^AB^** | 2824 | 2929 | 1368 |  | IV | 14270 | 14688 | 16758 | 19972 | 21304 | **26829^ABC^** |
|  | V | 7791 | 8471 | 6532 | 8799 | **12051^ABC^** | 9583 |  | V | 23518 | 24410 | 23803 | 29630 | 32229 | **40727^ABC^** |
| 9 | I | 4914 | **9379^ABC^** | 6623 | 7617 | 8131 | 7491 | 9 | I | 7499 | 6402 | **11189^ABC^** | 7515 | 8227 | 6634 |
|  | II | **9717^AC^** | 7811 | 7681 | 9224 | 8246 | 9580 |  | II | 3171 | 5399 | 6675 | 6420 | 9018 | **13767^AB^** |
|  | III | 6590 | 6263 | 7335 | 6167 | **8079^AB^** | 5458 |  | III | 2771 | 6434 | 6895 | 8472 | **10048^ABC^** | 9446 |
|  | IV | 7191 | 7693 | 9586 | 7241 | **10054^AC^** | 8027 |  | IV | 9140 | 9117 | 10785 | 9498 | 9586 | **15682^ABC^** |
|  | V | 4153 | 4792 | 5519 | 5697 | 5715 | **10393^AC^** |  | V | 12835 | 12382 | 13919 | 15984 | 15630 | **17192^ABC^** |
| 10 | I | 16919 | 19178 | 17940 | 16430 | 18067 | **22835^AC^** | 10 | I | 2452 | 489 | 731 | 1267 | 985 | **3910^C^** |
|  | II | 15185 | 14390 | 15661 | **19918^ABC^** | 17071 | 15546 |  | II | 1777 | 2719 | 1254 | 1588 | 2067 | **3219^BC^** |
|  | III | 13945 | **14767^ABC^** | 13652 | 11766 | 12458 | 12531 |  | III | 1131 | 1112 | **1669^B^** | 1510 | 1159 | 1566 |
|  | IV | 3872 | 4661 | 6114 | 7968 | 8693 | **9585^ABC^** |  | IV | 314 | 386 | 2162 | 4736 | 1885 | **8340^BC^** |
|  | V | 8563 | 7546 | 7806 | 4180 | **16274^ABC^** | 15861 |  | V | 2253 | 1484 | 2439 | 2616 | 3631 | **5507^C^** |
| 11 | I | 11714 | 13702 | **13723^ABC^** | 10306 | 10221 | 12110 | 11 | I | 866 | 841 | 1112 | 1694 | **1713^ABC^** | 1386 |
|  | II | 10637 | 13032 | **13588^AB^** | 11311 | 7933 | 12627 |  | II | 1184 | 1878 | 2418 | **2712^AB^** | 1778 | 2255 |
|  | III | 7193 | 7609 | 7205 | **8324^ABC^** | 6247 | 5648 |  | III | 2042 | 4512 | 4726 | **7139^ABC^** | 6631 | 4985 |
|  | IV | 6064 | 7534 | 9588 | 7006 | 8674 | **10654^ABC^** |  | IV | 2277 | 2964 | 2170 | 3306 | 3080 | **3893^ABC^** |
|  | V | 1996 | 1056 | 1667 | 2528 | **3367^ABC^** | 1108 |  | V | 3799 | 2781 | 2644 | 4061 | **6598^ABC^** | 4294 |
| 12 | I | 5237 | 5894 | 4099 | 9382 | 7450 | **9382^BC^** | 12 | I | 13315 | 7128 | 15657 | 14028 | **27419^ABC^** | 27351 |
|  | II | 4472 | 3394 | 5760 | **7779^BC^** | 5886 | 6016 |  | II | 15598 | 17802 | 22119 | 23307 | **29511^ABC^** | 27788 |
|  | III | 1990 | 3335 | 3210 | 4589 | **5410^ABC^** | 5133 |  | III | 5885 | 9180 | **15796^AC^** | 10216 | 7163 | 4240 |
|  | IV | 3365 | **4149^BC^** | 3448 | 4080 | 1691 | 1863 |  | IV | 13116 | 18515 | 18911 | **20140^ABC^** | 18362 | 12082 |
|  | V | 3854 | 5857 | 6440 | 10001 | **11664^AB^** | 8749 |  | V | 17350 | 15013 | 20022 | **23643^ABC^** | 21430 | 14559 |
| 13 | I | **7217^AB^** | 7215 | 6462 | 5739 | 3694 | 2854 | 13 | I | 6882 | 7237 | 6657 | 8012 | 8799 | **9290^ABC^** |
|  | II | 7875 | 6704 | 8267 | **9142^AB^** | 7803 | 7556 |  | II | 9490 | 10861 | 11801 | 13786 | **14890^ABC^** | 14635 |
|  | III | **6109^ABC^** | 4653 | 4383 | 5504 | 6046 | 5607 |  | III | 11322 | 13449 | 14521 | 15562 | 18040 | **18190^ABC^** |
|  | IV | 6890 | 7531 | **7746^AB^** | 4777 | 7482 | 5505 |  | IV | 8939 | 11373 | 11658 | 12163 | 12964 | **13249^BC^** |
|  | V | 6022 | 6512 | **6902^AB^** | 5649 | 4643 | 4805 |  | V | 6708 | 6609 | 9219 | 10484 | 9434 | **10689^ABC^** |
| 14 | I | **5193^AB^** | 1039 | 393 | 1680 | 780 | 1448 | 14 | I | 3577 | 2943 | 3093 | 3287 | 4376 | **6514^ABC^** |
|  | II | **1938^ABC^** | 510 | 464 | 235 | 384 | 422 |  | II | 3777 | 4636 | 5089 | 3451 | 5355 | **6191^AB^** |
|  | III | 1750 | 2398 | 2879 | 2365 | **3111^AC^** | 2861 |  | III | 2260 | 2535 | 2447 | 3167 | **4580^ABC^** | 3851 |
|  | IV | 1743 | **1767^ABC^** | 690 | 1051 | 357 | 622 |  | IV | 1525 | 2495 | 2405 | 3516 | **5385^ABC^** | 4355 |
|  | V | 1606 | 2007 | 1760 | **2136^BC^** | 2077 | 1070 |  | V | 1970 | 1841 | 2035 | 3475 | **4896^ABC^** | 4670 |
| 15 | I | 18937 | 17240 | 24472 | 26123 | 32829 | **33492^ABC^** | 15 | I | 1523 | 4116 | 3479 | 5853 | 2112 | **6496^ABC^** |
|  | II | 9733 | 10767 | 13990 | 15091 | 18645 | **23588^ABC^** |  | II | 8383 | 8226 | 5932 | **6906^ABC^** | 4006 | 6042 |
|  | III | 11065 | 16349 | 20127 | 18951 | 23792 | **25816^ABC^** |  | III | 5101 | 5663 | 5989 | 5310 | 6764 | **9800^ABC^** |
|  | IV | 16640 | 14889 | 29246 | 16660 | 29859 | **31579^C^** |  | IV | 4740 | 5559 | 5981 | 7041 | **9470^AC^** | 7943 |
|  | V | 14959 | 19033 | **21549^AB^** | 21079 | 20038 | 16200 |  | V | 4156 | **4984^AC^** | 4420 | 4090 | 4080 | 3565 |
| 16 | I | 8884 | 11207 | 7968 | **11905^AC^** | 6173 | 6470 | 16 | I | 2591 | 2261 | 2499 | **5810^ABC^** | 2536 | 3593 |
|  | II | **7788^ABC^** | 5669 | 3924 | 1582 | 2380 | 5059 |  | II | 5287 | 5360 | 5380 | 5815 | **7276^ABC^** | 6349 |
|  | III | **4458^ABC^** | 2444 | 1170 | 2472 | 3408 | 1513 |  | III | 4243 | 4688 | 4473 | **6029^AC^** | 5335 | 4480 |
|  | IV | 798 | 571 | 1612 | 1512 | **4443^ABC^** | 3417 |  | IV | 1475 | 1767 | 3068 | 2228 | **4208^ABC^** | 1909 |
|  | V | **2496^AC^** | 2223 | 1060 | 201 | 1479 | 2198 |  | V | 4677 | 3975 | 4503 | **6508^ABC^** | 4485 | 3521 |
| 17 | I | 7180 | 13213 | 8634 | **18629^ABC^** | 9644 | 9744 | 17 | I | 6436 | 8451 | **8679^AB^** | 7444 | 5770 | 4814 |
|  | II | 12807 | 14133 | 13247 | 15307 | 6770 | **17271^C^** |  | II | 4893 | **6061^ABC^** | 3879 | 3329 | 2195 | 2530 |
|  | III | 8059 | 7602 | 6435 | 12060 | **16780^B^** | 9257 |  | III | 3429 | 1514 | 2830 | 2736 | **3530^ABC^** | 1383 |
|  | IV | 10278 | 5661 | 4852 | 4737 | **12381^ABC^** | 8470 |  | IV | 3947 | **5389^C^** | 3829 | 3815 | 3832 | 4931 |
|  | V | **10650^A^** | 9634 | 9865^ABC^ | 9644 | 7836 | 10097 |  | V | 5355 | 6803 | 7890 | **7893^ABC^** | 7306 | 5969 |
| 18 | I | 10734 | 11709 | 13895 | 15686 | **16228^ABC^** | 12402 | 18 | I | 9816 | 14019 | 11080 | **19217^ABC^** | 16283 | 14222 |
|  | II | 9614 | 9459 | 11805 | 12039 | **16148^ABC^** | 14061 |  | II | 7766 | 5763 | 8721 | 6265 | **9560^AB^** | 8923 |
|  | III | 5551 | 9010 | 7213 | **10159^ABC^** | 9900 | 9102 |  | III | 12383 | 12867 | 12069 | **16588^BC^** | 13382 | 7802 |
|  | IV | 9023 | 9142 | 9821 | 8948 | **14002^ABC^** | 12733 |  | IV | 5489 | 5266 | **6353^ABC^** | 5338 | 3884 | 5089 |
|  | V | 13599 | 14101 | 13871 | 15386 | **18308^ABC^** | 11565 |  | V | 1409 | 1087 | 1604 | 2382 | **3680^ABC^** | 3266 |
| 19 | I | 13411 | 11678 | 13118 | 11003 | **15338^ABC^** | 9493 | 19 | I | 6105 | **8325^ABC^** | 6649 | 6505 | 7262 | 6584 |
|  | II | **13020^AB^** | 8527 | 8463 | 8974 | 7735 | 6169 |  | II | 1084 | **1734^B^** | 425 | 940 | 868 | 719 |
|  | III | 4633 | 5822 | 9915 | 9263 | 9666 | **12283^ABC^** |  | III | 1667 | 1587 | 1793 | **2203^AB^** | 2166 | 1956 |
|  | IV | **6783^ABC^** | 6388 | 3957 | 3517 | 5941 | 6541 |  | IV | 2403 | 2161 | 1964 | **2754^BC^** | 2543 | 2512 |
|  | V | **16872^ABC^** | 14404 | 16192 | 12900 | 15437 | 8841 |  | V | 2426 | 2409 | 2766 | 2925 | 3956 | **3997^ABC^** |
| 20 | I | 3903 | 5498 | 5680 | 5105 | **7053^AC^** | 3891 | 20 | I | 6463 | 7278 | 6832 | **8088^ABC^** | 7593 | 8058 |
|  | II | 2965 | 2640 | 3492 | 4866 | 5627 | **7055^ABC^** |  | II | 1984 | 2650 | 2614 | 3851 | **4746^ABC^** | 4550 |
|  | III | 4596 | 6431 | 7742 | 6504 | 6735 | **9543^ABC^** |  | III | 6661 | 6335 | 7900 | **8068^AC^** | 7339 | 7557 |
|  | IV | 1984 | 2650 | 2614 | 3851 | **4746^ABC^** | 4550 |  | IV | 7059 | 7094 | 7167 | 7511 | 8022 | **8275^ABC^** |
|  | V | 2462 | 2101 | 2921 | 2266 | 3883 | **4908^ABC^** |  | V | 6238 | 7700 | 7971 | 8497 | 8417 | **8762^ABC^** |
| 21 | I | 3567 | 3684 | 6455 | 6931 | 6769 | **8261^BC^** | 21 | I | 5710 | 3618 | 3079 | 3159 | 5045 | **6105^AC^** |
|  | II | 5734 | 5301 | **7335^AC^** | 5058 | 5107 | 3905 |  | II | 1986 | 1657 | 880 | 2459 | **2617^ABC^** | 2093 |
|  | III | 3726 | 4669 | 4793 | 3405 | 4159 | **5275^A^** |  | III | 1375 | 1038 | **2806^C^** | 1319 | 1330 | 2459 |
|  | IV | 3072 | 1937 | **5711^AC^** | 3851 | 2922 | 2364 |  | IV | 373 | 170 | 96 | 200 | 257 | **664** |
|  | V | 2697 | **5163^ABC^** | 4894 | 3660 | 4007 | 4875 |  | V | 1499 | 1203 | 393 | 168 | 93 | **1520^ABC^** |
| 22 | I | 9910 | **14508^ABC^** | 11277 | 11977 | 13072 | 11612 | 22 | I | 6314 | 6614 | **7444^AB^** | 6283 | 6949 | 6628 |
|  | II | 12104 | 12472 | 15518 | 14479 | 12787 | **15915^AC^** |  | II | 2582 | 3539 | 4783 | 5314 | **5352^ABC^** | 5218 |
|  | III | 10898 | 13507 | 14881 | 15470 | **16581^BC^** | 16554 |  | III | 2533 | 2886 | **3019^ABC^** | 2864 | 1055 | 1076 |
|  | IV | 9966 | **14406^AC^** | 10276 | 12424 | 10939 | 13093 |  | IV | 1663 | 2554 | 3330 | 4118 | **5993^ABC^** | 5850 |
|  | V | 3782 | 7504 | 8202 | 7983 | 11088 | **11202^BC^** |  | V | **1535^AB^** | 613 | 429 | 265 | 423 | 146 |
| 23 | I | 7075 | 6536 | 8750 | 6840 | **9042^AB^** | 7503 | 23 | I | 6692 | 7906 | 5781 | **10610^ABC^** | 9966 | 8418 |
|  | II | **11666^AB^** | 9910 | 10624 | 9754 | 3216 | 8999 |  | II | 2531 | 5174 | 3099 | **5177^ABC^** | 5977 | 5022 |
|  | III | 9573 | **9987^ABC^** | 9236 | 7544 | 6517 | 85337 |  | III | 2634 | 5013 | 3844 | 3503 | 4149 | **5752^ABC^** |
|  | IV | 8825 | 8139 | 7163 | **8985^ABC^** | 6303 | 5141 |  | IV | 2055 | 3018 | **4463^ABC^** | 4135 | 3775 | 3554 |
|  | V | 8300 | 8097 | 7867 | 6127 | **9841^AC^** | 5963 |  | V | 3495 | 7654 | 3952 | 6532 | **9292^ABC^** | 6984 |
| 24 | I | 5152 | 6508 | **7553^ABC^** | 6066 | 5401 | 4453 | 24 | I | 1570 | 787 | 2183 | 3348 | 5715 | **10729^ABC^** |
|  | II | **10158^AB^** | 8834 | 8429 | 7940 | 7114 | 7963 |  | II | 1271 | 1763 | 4571 | **4908^ABC^** | 4042 | 4273 |
|  | III | 11290 | 8469 | 11738 | 11648 | 11868 | **13022^ABC^** |  | III | 750 | 940 | 1194 | 591 | **1690^B^** | 686 |
|  | IV | 5732 | 6612 | 6294 | 6179 | 10439 | **11127^ABC^** |  | IV | 2643 | 3204 | 2927 | **4153^ABC^** | 3939 | 3598 |
|  | V | 11737 | 11233 | 11407 | 10826 | 12064 | **13202^ABC^** |  | V | 2556 | 3127 | 3052 | **4209^ABC^** | 4142 | 3362 |
| 25 | I | 1802 | 1857 | 1767 | 2650 | 2574 | **2918^BC^** | 25 | I | 4824 | 8125 | 7534 | **11121^ABC^** | 8774 | 8223 |
|  | II | 922 | 673 | 1039 | 1259 | 1469 | **1727^BC^** |  | II | 7731 | 7979 | 7408 | 8789 | **9734^ABC^** | 9265 |
|  | III | **2158^AB^** | 1370 | 1553 | 2118 | 2138 | 2001 |  | III | 4831 | 5329 | 6481 | **12205^ABC^** | 8958 | 7185 |
|  | IV | 1016 | 638 | 772 | 605 | 932 | **1656^BC^** |  | IV | **12534^ABC^** | 8241 | 8515 | 5849 | 5228 | 8047 |
|  | V | 568 | 1559 | 1464 | **2190^ABC^** | 2080 | 2162 |  | V | 1859 | 4071 | 1952 | 1889 | 2745 | **5728^ABC^** |
| 26 | I | 16503 | 16486 | 12443 | 17751 | 16337 | **18844^AB^** | 26 | I | 17141 | 15877 | 17101 | **21628^ABC^** | 21396 | 20056 |
|  | II | **25298^A^** | 24799 | 20066 | 22784 | 24471 | 23124 |  | II | 15521 | 16227 | 13177 | 17335 | 19508 | **24806^ABC^** |
|  | III | 17940 | **26106^ABC^** | 23355 | 22866 | 21475 | 23070 |  | III | 10440 | 18508 | 19304 | 21905 | 20093 | **25364^AC^** |
|  | IV | 17274 | 13704 | 19866 | 20659 | 22198 | **25111^ABC^** |  | IV | 12825 | 18213 | 19205 | 23484 | 22685 | **31517^ABC^** |
|  | V | 25212 | 24496 | **29584^AC^** | 23598 | 29457 | 25884 |  | V | 16643 | 19767 | 20893 | 23269 | **31812^ABC^** | 27229 |

| 0.1 HZ Group | | | | | | | | RF Group | | | | | | | |
| --- | --- | --- | --- | --- | --- | --- | --- | --- | --- | --- | --- | --- | --- | --- | --- |
| No | **Absolute power in the LF band [ms²]** | | | | | | **No** | | **Absolute power in the LF band [ms²]** | | | | | |  |
|  | **7.0 bpm** | **6.5 bpm** | **6.0 bpm** | **5.5 bpm** | **5.0 bpm** | **4.5 bpm** |  |  | **7.0 bpm** | **6.5 bpm** | **6.0 bpm** | **5.5 bpm** | **5.0 bpm** | **4.5 bpm** |  |
| 1 | . | 15250 | 14225 | 14324 | **15954^AB^** | 14811 | **1** | | 12768 | **13418^ABC^** | 12530 | 9993 | 8061 | 4455 |  |
|  | 9856 | 14132 | 8518 | 13620 | 14759 | **14907^A^** |  |  | 6065 | 6076 | 3204 | 4099 | 2326 | **8252^A^** |  |
|  | 4984 | 12219 | 10465 | 10937 | 14127 | **14426^ABC^** |  |  | 2023 | 2541 | **4389^AB^** | 2285 | 3943 | 2583 |  |
|  | 10771 | 12261 | 12586 | 13290 | **14642^AB^** | 13027 |  |  | 4102 | 2901 | **6623^AB^** | 4707 | 5795 | 1003 |  |
|  | 5739 | 6812 | 9293 | 9879 | **14442^ABC^** | 11543 |  |  | **4963^ABC^** | 2758 | 2473 | 1124 | 825 | 1073 |  |
| 2 | 1974 | 3418 | 1972 | **3874^ABC^** | 3108 | 3419 | **2** | | 6034 | 4985 | 5415 | **6564^AC^** | 6437 | 3114 |  |
|  | 3563 | **3777^AC^** | 2956 | 3496 | 2344 | . |  |  | **5946^A^** | . | . | . | . | . |  |
|  | 2246 | 2322 | **5135^AB^** | 4221 | 3796 | 3175 |  |  | 5602 | 5768 | 4062 | **6475^AC^** | 6032 | 6237 |  |
|  | 1906 | 2732 | **3523^AB^** | 2927 | 1244 | 1392 |  |  | 2452 | 3444 | 5337 | 4568 | **5465^ABC^** | 3391 |  |
|  | 2425 | 3344 | 2521 | 2928 | 3729 | **5806^ABC^** |  |  | 3211 | 4239 | 3321 | **4756^ABC^** | 4044 | 4790 |  |
| 3 | 9206 | **16065^ABC^** | 15396 | 8550 | . | . | **3** | | 4473 | 4895 | 4225 | 5411 | 6092 | **7578^ABC^** |  |
|  | **11187^ABC^** | 10761 | 8053 | 9945 | 6517 | 6372 |  |  | 2453 | 3812 | 3659 | 4308 | 4398 | **4879^ABC^** |  |
|  | . | . | . | . | . | **3001^AB^** |  |  | 3633 | 3697 | **6074^AB^** | 2979 | 4662 | 2854 |  |
|  | 4200 | 5262 | **8984** | 5574 | 5246 | 6724 |  |  | 3470 | 3401 | **5083^A^** | 5011 | 2734 | 4772 |  |
|  | 2816 | 3015 | 3879 | 4626 | **4858^ABC^** | 4820 |  |  | 4015 | 3855 | 4959 | 6726 | **7532^ABC^** | 5933 |  |
| 4 | 4015 | 3769 | 9731 | 4774 | 5061 | **6653^B^** | **4** | | 1288 | 1955 | 1472 | 911 | **2147^AC^** | 1993 |  |
|  | 4369 | 5411 | 5729 | 7282 | 8839 | **11430^ABC^** |  |  | 938 | 1490 | 1608 | 1538 | 1962^ABC^ | **2059^ABC^** |  |
|  | 5073 | 6403 | 6355 | 7489 | **9062^ABC^** | 9054 |  |  | 721 | 1183 | 1626 | **1676^AB^** | 994 | 1352 |  |
|  | 9414 | 9484 | 10522 | 10112 | 13107 | **16071^ABC^** |  |  | **18193** | 17447 | 13821 | 15641 | 17660 | 16243 |  |
|  | 7415 | 6123 | 6694 | 7856 | 9247 | **10062^ABC^** |  |  | 1140 | 1258 | 1950^ABC^ | 1408 | **1985^ABC^** | 1619 |  |
| 5 | **16224^AB^** | 13926 | 14949 | 13999 | 14967 | 14732 | **5** | | 3948 | 3105 | 3130 | 4340 | 6910 | **6962^BC^** |  |
|  | 9744 | 13235 | 17011 | 16197 | 17349 | **21451^ABC^** |  |  | **2297^A^** | 1768 | 1878 | 1481 | 934 | 1417 |  |
|  | 16003 | 15503 | 15385 | 16074 | 13654 | **20312^ABC^** |  |  | 5286 | 2326 | 5183 | 3328 | 5005 | **5307^BC^** |  |
|  | **18193^B^** | 17447 | 13821 | 15641 | 17660 | 16243 |  |  | 7526 | 8420 | **11034^AB^** | 7434 | 6740 | 8212 |  |
|  | 12934 | 15471 | 15187 | 16772 | 18967 | **20147^ABC^** |  |  | **10532^ABC^** | 6033 | 7236 | 7102 | 7174 | 7593 |  |
| 6 | 8340 | 11213 | 9117 | 8757 | 14838 | **19383^AC^** | **6** | | 1166 | 1676 | 1266 | 4088 | **7314^A^** | 4778 |  |
|  | 1709 | 1214 | 2027 | 2548 | 3530 | **6574^ABC^** |  |  | 1362 | 3538 | **3627^ABC^** | 2680 | 2904 | 3301 |  |
|  | 2365 | **2467^C^** | 1836 | 2150 | 2115 | 1830 |  |  | 304 | 1014 | **2811^ABC^** | 2543 | 1616 | 1967 |  |
|  | 2337 | 3917 | 5899 | **6038^AC^** | 5496 | 4748 |  |  | 1515 | 1654 | 1740 | 1989 | 2252 | **2880^ABC^** |  |
|  | 659 | 958 | 1637 | 1867 | 944 | **4719^AC^** |  |  | 1730 | 3575 | 3154 | 3242 | **4400^ABC^** | 2735 |  |
| 7 | 3209 | 4526 | 1981 | **16237^ABC^** | 1042 | 6035 | **7** | | **4718^AC^** | 2979 | 2030 | 1600 | 1506 | 2763 |  |
|  | 5112 | 4542 | 7128 | 9348 | 9319 | **11118^C^** |  |  | 2293 | 1967 | 2518 | 2368 | **2638^C^** | 1824 |  |
|  | 10727 | 7148 | 13268 | **15967^ABC^** | 12727 | 12930 |  |  | **1836^AC^** | 705 | 650 | 1076 | 72 | 1118 |  |
|  | 8269 | 7065 | 9353 | 11489 | 10430 | **14206^AB^** |  |  | **3189^AB^** | 1715 | 2367 | 1343 | 1017 | 1629 |  |
|  | 1215 | 2275 | 5729 | **9838^AC^** | 5952 | 8505 |  |  | **7674^A^** | 5826 | 4259 | 5778 | 2458 | 4081 |  |
| 8 | 12729 | 12395 | 10023 | 12666 | 11727 | **13279^ABC^** | **8** | | 18346 | 15982 | 18662 | 19069 | 20753 | **23573^ABC^** |  |
|  | 1329 | 1319 | 1571 | 2248 | 2421 | **2961^ABC^** |  |  | 21389 | 18393 | 20394 | 23856 | 20962 | **29050^ABC^** |  |
|  | 472 | 487 | 642 | 1708 | 1959 | **2760^ABC^** |  |  | 18533 | 21155 | 19723 | 27135 | **29973^AC^** | 26167 |  |
|  | 616 | 1043 | **3002^AB^** | 2824 | 2929 | 1368 |  |  | 14270 | 14688 | 16758 | 19972 | 21304 | **26829^ABC^** |  |
|  | 7791 | 8471 | 6532 | 8799 | **12051^ABC^** | 9583 |  |  | 23518 | 24410 | 23803 | 29630 | 32229 | **40727^ABC^** |  |
| 9 | 4914 | **9379^ABC^** | 6623 | 7617 | 8131 | 7491 | **9** | | 7499 | 6402 | **11189^ABC^** | 7515 | 8227 | 6634 |  |
|  | **9717^AC^** | 7811 | 7681 | 9224 | 8246 | 9580 |  |  | 3171 | 5399 | 6675 | 6420 | 9018 | **13767^AB^** |  |
|  | 6590 | 6263 | 7335 | 6167 | **8079^AB^** | 5458 |  |  | 2771 | 6434 | 6895 | 8472 | **10048^ABC^** | 9446 |  |
|  | 7191 | 7693 | 9586 | 7241 | **10054^AC^** | 8027 |  |  | 9140 | 9117 | 10785 | 9498 | 9586 | **15682^ABC^** |  |
|  | 4153 | 4792 | 5519 | 5697 | 5715 | **10393^AC^** |  |  | 12835 | 12382 | 13919 | 15984 | 15630 | **17192^ABC^** |  |
| 10 | 16919 | 19178 | 17940 | 16430 | 18067 | **22835^AC^** | **10** | | 2452 | 489 | 731 | 1267 | 985 | **3910^C^** |  |
|  | 15185 | 14390 | 15661 | **19918^ABC^** | 17071 | 15546 |  |  | 1777 | 2719 | 1254 | 1588 | 2067 | **3219^BC^** |  |
|  | 13945 | **14767^ABC^** | 13652 | 11766 | 12458 | 12531 |  |  | 1131 | 1112 | **1669^B^** | 1510 | 1159 | 1566 |  |
|  | 3872 | 4661 | 6114 | 7968 | 8693 | **9585^ABC^** |  |  | 314 | 386 | 2162 | 4736 | 1885 | **8340^BC^** |  |
|  | 8563 | 7546 | 7806 | 4180 | **16274^ABC^** | 15861 |  |  | 2253 | 1484 | 2439 | 2616 | 3631 | **5507^C^** |  |
| 11 | 11714 | 13702 | **13723^ABC^** | 10306 | 10221 | 12110 | **11** | | 866 | 841 | 1112 | 1694 | **1713^ABC^** | 1386 |  |
|  | 10637 | 13032 | **13588^AB^** | 11311 | 7933 | 12627 |  |  | 1184 | 1878 | 2418 | **2712^AB^** | 1778 | 2255 |  |
|  | 7193 | 7609 | 7205 | **8324^ABC^** | 6247 | 5648 |  |  | 2042 | 4512 | 4726 | **7139^ABC^** | 6631 | 4985 |  |
|  | 6064 | 7534 | 9588 | 7006 | 8674 | **10654^ABC^** |  |  | 2277 | 2964 | 2170 | 3306 | 3080 | **3893^ABC^** |  |
|  | 1996 | 1056 | 1667 | 2528 | **3367^ABC^** | 1108 |  |  | 3799 | 2781 | 2644 | 4061 | **6598^ABC^** | 4294 |  |
| 12 | 5237 | 5894 | 4099 | 9382 | 7450 | **9382^BC^** | **12** | | 13315 | 7128 | 15657 | 14028 | **27419^ABC^** | 27351 |  |
|  | 4472 | 3394 | 5760 | **7779^BC^** | 5886 | 6016 |  |  | 15598 | 17802 | 22119 | 23307 | **29511^ABC^** | 27788 |  |
|  | 1990 | 3335 | 3210 | 4589 | **5410^ABC^** | 5133 |  |  | 5885 | 9180 | **15796^AC^** | 10216 | 7163 | 4240 |  |
|  | 3365 | **4149^BC^** | 3448 | 4080 | 1691 | 1863 |  |  | 13116 | 18515 | 18911 | **20140^ABC^** | 18362 | 12082 |  |
|  | 3854 | 5857 | 6440 | 10001 | **11664^AB^** | 8749 |  |  | 17350 | 15013 | 20022 | **23643^ABC^** | 21430 | 14559 |  |
| 13 | **7217^AB^** | 7215 | 6462 | 5739 | 3694 | 2854 | **13** | | 6882 | 7237 | 6657 | 8012 | 8799 | **9290^ABC^** |  |
|  | 7875 | 6704 | 8267 | **9142^AB^** | 7803 | 7556 |  |  | 9490 | 10861 | 11801 | 13786 | **14890^ABC^** | 14635 |  |
|  | **6109^ABC^** | 4653 | 4383 | 5504 | 6046 | 5607 |  |  | 11322 | 13449 | 14521 | 15562 | 18040 | **18190^ABC^** |  |
|  | 6890 | 7531 | **7746^AB^** | 4777 | 7482 | 5505 |  |  | 8939 | 11373 | 11658 | 12163 | 12964 | **13249^BC^** |  |
|  | 6022 | 6512 | **6902^AB^** | 5649 | 4643 | 4805 |  |  | 6708 | 6609 | 9219 | 10484 | 9434 | **10689^ABC^** |  |
| 14 | **5193^AB^** | 1039 | 393 | 1680 | 780 | 1448 | **14** | | 3577 | 2943 | 3093 | 3287 | 4376 | **6514^ABC^** |  |
|  | **1938^ABC^** | 510 | 464 | 235 | 384 | 422 |  |  | 3777 | 4636 | 5089 | 3451 | 5355 | **6191^AB^** |  |
|  | 1750 | 2398 | 2879 | 2365 | **3111^AC^** | 2861 |  |  | 2260 | 2535 | 2447 | 3167 | **4580^ABC^** | 3851 |  |
|  | 1743 | **1767^ABC^** | 690 | 1051 | 357 | 622 |  |  | 1525 | 2495 | 2405 | 3516 | **5385^ABC^** | 4355 |  |
|  | 1606 | 2007 | 1760 | **2136^BC^** | 2077 | 1070 |  |  | 1970 | 1841 | 2035 | 3475 | **4896^ABC^** | 4670 |  |
| 15 | 18937 | 17240 | 24472 | 26123 | 32829 | **33492^ABC^** | **15** | | 1523 | 4116 | 3479 | 5853 | 2112 | **6496^ABC^** |  |
|  | 9733 | 10767 | 13990 | 15091 | 18645 | **23588^ABC^** |  |  | 8383 | 8226 | 5932 | **6906^ABC^** | 4006 | 6042 |  |
|  | 11065 | 16349 | 20127 | 18951 | 23792 | **25816^ABC^** |  |  | 5101 | 5663 | 5989 | 5310 | 6764 | **9800^ABC^** |  |
|  | 16640 | 14889 | 29246 | 16660 | 29859 | **31579^C^** |  |  | 4740 | 5559 | 5981 | 7041 | **9470^AC^** | 7943 |  |
|  | 14959 | 19033 | **21549^AB^** | 21079 | 20038 | 16200 |  |  | 4156 | **4984^AC^** | 4420 | 4090 | 4080 | 3565 |  |
| 16 | 8884 | 11207 | 7968 | **11905^AC^** | 6173 | 6470 | **16** | | 2591 | 2261 | 2499 | **5810^ABC^** | 2536 | 3593 |  |
|  | **7788^ABC^** | 5669 | 3924 | 1582 | 2380 | 5059 |  |  | 5287 | 5360 | 5380 | 5815 | **7276^ABC^** | 6349 |  |
|  | **4458^ABC^** | 2444 | 1170 | 2472 | 3408 | 1513 |  |  | 4243 | 4688 | 4473 | **6029^AC^** | 5335 | 4480 |  |
|  | 798 | 571 | 1612 | 1512 | **4443^ABC^** | 3417 |  |  | 1475 | 1767 | 3068 | 2228 | **4208^ABC^** | 1909 |  |
|  | **2496^AC^** | 2223 | 1060 | 201 | 1479 | 2198 |  |  | 4677 | 3975 | 4503 | **6508^ABC^** | 4485 | 3521 |  |
| 17 | 7180 | 13213 | 8634 | **18629^ABC^** | 9644 | 9744 | **17** | | 6436 | 8451 | **8679^AB^** | 7444 | 5770 | 4814 |  |
|  | 12807 | 14133 | 13247 | 15307 | 6770 | **17271^C^** |  |  | 4893 | **6061^ABC^** | 3879 | 3329 | 2195 | 2530 |  |
|  | 8059 | 7602 | 6435 | 12060 | **16780^B^** | 9257 |  |  | 3429 | 1514 | 2830 | 2736 | **3530^ABC^** | 1383 |  |
|  | 10278 | 5661 | 4852 | 4737 | **12381^ABC^** | 8470 |  |  | 3947 | **5389^C^** | 3829 | 3815 | 3832 | 4931 |  |
|  | **10650^A^** | 9634 | 9865^ABC^ | 9644 | 7836 | 10097 |  |  | 5355 | 6803 | 7890 | **7893^ABC^** | 7306 | 5969 |  |
| 18 | 10734 | 11709 | 13895 | 15686 | **16228^ABC^** | 12402 | **18** | | 9816 | 14019 | 11080 | **19217^ABC^** | 16283 | 14222 |  |
|  | 9614 | 9459 | 11805 | 12039 | **16148^ABC^** | 14061 |  |  | 7766 | 5763 | 8721 | 6265 | **9560^AB^** | 8923 |  |
|  | 5551 | 9010 | 7213 | **10159^ABC^** | 9900 | 9102 |  |  | 12383 | 12867 | 12069 | **16588^BC^** | 13382 | 7802 |  |
|  | 9023 | 9142 | 9821 | 8948 | **14002^ABC^** | 12733 |  |  | 5489 | 5266 | **6353^ABC^** | 5338 | 3884 | 5089 |  |
|  | 13599 | 14101 | 13871 | 15386 | **18308^ABC^** | 11565 |  |  | 1409 | 1087 | 1604 | 2382 | **3680^ABC^** | 3266 |  |
| 19 | 13411 | 11678 | 13118 | 11003 | **15338^ABC^** | 9493 | **19** | | 6105 | **8325^ABC^** | 6649 | 6505 | 7262 | 6584 |  |
|  | **13020^AB^** | 8527 | 8463 | 8974 | 7735 | 6169 |  |  | 1084 | **1734^B^** | 425 | 940 | 868 | 719 |  |
|  | 4633 | 5822 | 9915 | 9263 | 9666 | **12283^ABC^** |  |  | 1667 | 1587 | 1793 | **2203^AB^** | 2166 | 1956 |  |
|  | **6783^ABC^** | 6388 | 3957 | 3517 | 5941 | 6541 |  |  | 2403 | 2161 | 1964 | **2754^BC^** | 2543 | 2512 |  |
|  | **16872^ABC^** | 14404 | 16192 | 12900 | 15437 | 8841 |  |  | 2426 | 2409 | 2766 | 2925 | 3956 | **3997^ABC^** |  |
| 20 | 3903 | 5498 | 5680 | 5105 | **7053^AC^** | 3891 | **20** | | 6463 | 7278 | 6832 | **8088^ABC^** | 7593 | 8058 |  |
|  | 2965 | 2640 | 3492 | 4866 | 5627 | **7055^ABC^** |  |  | 1984 | 2650 | 2614 | 3851 | **4746^ABC^** | 4550 |  |
|  | 4596 | 6431 | 7742 | 6504 | 6735 | **9543^ABC^** |  |  | 6661 | 6335 | 7900 | **8068^AC^** | 7339 | 7557 |  |
|  | 1984 | 2650 | 2614 | 3851 | **4746^ABC^** | 4550 |  |  | 7059 | 7094 | 7167 | 7511 | 8022 | **8275^ABC^** |  |
|  | 2462 | 2101 | 2921 | 2266 | 3883 | **4908^ABC^** |  |  | 6238 | 7700 | 7971 | 8497 | 8417 | **8762^ABC^** |  |
| 21 | 3567 | 3684 | 6455 | 6931 | 6769 | **8261^BC^** | **21** | | 5710 | 3618 | 3079 | 3159 | 5045 | **6105^AC^** |  |
|  | 5734 | 5301 | **7335^AC^** | 5058 | 5107 | 3905 |  |  | 1986 | 1657 | 880 | 2459 | **2617^ABC^** | 2093 |  |
|  | 3726 | 4669 | 4793 | 3405 | 4159 | **5275^A^** |  |  | 1375 | 1038 | **2806^C^** | 1319 | 1330 | 2459 |  |
|  | 3072 | 1937 | **5711^AC^** | 3851 | 2922 | 2364 |  |  | 373 | 170 | 96 | 200 | 257 | **664** |  |
|  | 2697 | **5163^ABC^** | 4894 | 3660 | 4007 | 4875 |  |  | 1499 | 1203 | 393 | 168 | 93 | **1520^ABC^** |  |
| 22 | 9910 | **14508^ABC^** | 11277 | 11977 | 13072 | 11612 | **22** | | 6314 | 6614 | **7444^AB^** | 6283 | 6949 | 6628 |  |
|  | 12104 | 12472 | 15518 | 14479 | 12787 | **15915^AC^** |  |  | 2582 | 3539 | 4783 | 5314 | **5352^ABC^** | 5218 |  |
|  | 10898 | 13507 | 14881 | 15470 | **16581^BC^** | 16554 |  |  | 2533 | 2886 | **3019^ABC^** | 2864 | 1055 | 1076 |  |
|  | 9966 | **14406^AC^** | 10276 | 12424 | 10939 | 13093 |  |  | 1663 | 2554 | 3330 | 4118 | **5993^ABC^** | 5850 |  |
|  | 3782 | 7504 | 8202 | 7983 | 11088 | **11202^BC^** |  |  | **1535^AB^** | 613 | 429 | 265 | 423 | 146 |  |
| 23 | 7075 | 6536 | 8750 | 6840 | **9042^AB^** | 7503 | **23** | | 6692 | 7906 | 5781 | **10610^ABC^** | 9966 | 8418 |  |
|  | **11666^AB^** | 9910 | 10624 | 9754 | 3216 | 8999 |  |  | 2531 | 5174 | 3099 | **5177^ABC^** | 5977 | 5022 |  |
|  | 9573 | **9987^ABC^** | 9236 | 7544 | 6517 | 85337 |  |  | 2634 | 5013 | 3844 | 3503 | 4149 | **5752^ABC^** |  |
|  | 8825 | 8139 | 7163 | **8985^ABC^** | 6303 | 5141 |  |  | 2055 | 3018 | **4463^ABC^** | 4135 | 3775 | 3554 |  |
|  | 8300 | 8097 | 7867 | 6127 | **9841^AC^** | 5963 |  |  | 3495 | 7654 | 3952 | 6532 | **9292^ABC^** | 6984 |  |
| 24 | 5152 | 6508 | **7553^ABC^** | 6066 | 5401 | 4453 | **24** | | 1570 | 787 | 2183 | 3348 | 5715 | **10729^ABC^** |  |
|  | **10158^AB^** | 8834 | 8429 | 7940 | 7114 | 7963 |  |  | 1271 | 1763 | 4571 | **4908^ABC^** | 4042 | 4273 |  |
|  | 11290 | 8469 | 11738 | 11648 | 11868 | **13022^ABC^** |  |  | 750 | 940 | 1194 | 591 | **1690^B^** | 686 |  |
|  | 5732 | 6612 | 6294 | 6179 | 10439 | **11127^ABC^** |  |  | 2643 | 3204 | 2927 | **4153^ABC^** | 3939 | 3598 |  |
|  | 11737 | 11233 | 11407 | 10826 | 12064 | **13202^ABC^** |  |  | 2556 | 3127 | 3052 | **4209^ABC^** | 4142 | 3362 |  |
| 25 | 1802 | 1857 | 1767 | 2650 | 2574 | **2918^BC^** | **25** | | 4824 | 8125 | 7534 | **11121^ABC^** | 8774 | 8223 |  |
|  | 922 | 673 | 1039 | 1259 | 1469 | **1727^BC^** |  |  | 7731 | 7979 | 7408 | 8789 | **9734^ABC^** | 9265 |  |
|  | **2158^AB^** | 1370 | 1553 | 2118 | 2138 | 2001 |  |  | 4831 | 5329 | 6481 | **12205^ABC^** | 8958 | 7185 |  |
|  | 1016 | 638 | 772 | 605 | 932 | **1656^BC^** |  |  | **12534^ABC^** | 8241 | 8515 | 5849 | 5228 | 8047 |  |
|  | 568 | 1559 | 1464 | **2190^ABC^** | 2080 | 2162 |  |  | 1859 | 4071 | 1952 | 1889 | 2745 | **5728^ABC^** |  |
| 26 | 16503 | 16486 | 12443 | 17751 | 16337 | **18844^AB^** | **26** | | 17141 | 15877 | 17101 | **21628^ABC^** | 21396 | 20056 |  |
|  | **25298^A^** | 24799 | 20066 | 22784 | 24471 | 23124 |  |  | 15521 | 16227 | 13177 | 17335 | 19508 | **24806^ABC^** |  |
|  | 17940 | **26106^ABC^** | 23355 | 22866 | 21475 | 23070 |  |  | 10440 | 18508 | 19304 | 21905 | 20093 | **25364^AC^** |  |
|  | 17274 | 13704 | 19866 | 20659 | 22198 | **25111^ABC^** |  |  | 12825 | 18213 | 19205 | 23484 | 22685 | **31517^ABC^** |  |
|  | 25212 | 24496 | **29584^AC^** | 23598 | 29457 | 25884 |  |  | 16643 | 19767 | 20893 | 23269 | **31812^ABC^** | 27229 |  |

Note. Letters indicate that the following criteria for RF determination were met: A = phase synchrony between the respirometer and heart rate waveforms; B = peak-to-trough amplitude (HRmax – HRmin); C = maximum LF amplitude peak..
