# Supplementary material for: Resonance frequency versus fixed 0.1 Hz breathing in HRV biofeedback: a four-week randomized comparison
Source: Sci Rep. 2026 May 19;16:22630. doi: 10.1038/s41598-026-53333-6 (PMC13381940; doi:10.1038/s41598-026-53333-6)
Supplement: Supplementary file 2 — Supplementary Material 2 [file 41598_2026_53333_MOESM2_ESM.docx]

**Supplement 1. Structure of the Home-Based Slow-Paced Breathing Training**

The structure of Home-Based Slow-Paced Breathing Training is presented below, along with instructions for participants.

**Weekly breathing practice**

“A week of breathing practice is ahead of you. In the materials, you will find a video that will help you perform breathing exercises at a slow and relaxed pace. Remember to practice breathing at least once a day for 20 minutes.

Find time for yourself to relax. Remember not to let anyone disturb you during your practice. This is a time just for you. Remember to practice every day to achieve the beneficial effects of the breathing exercises. If you feel unwell during this exercise, remember to stop the exercise and inform us of the situation”.

**Mood assessment before the breathing exercise**

“Before you begin the exercise, please rate how you feel right now on a scale. Mark the dot that is closest to the adjective that describes how you feel at this moment”.


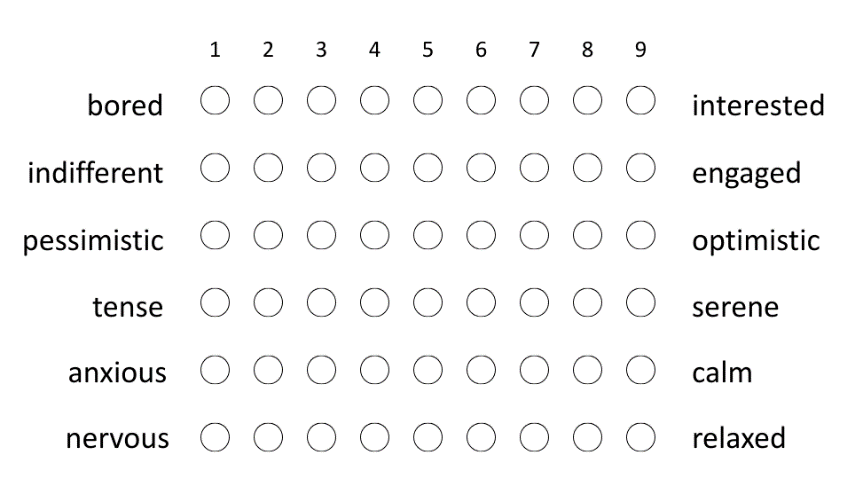


**Figure 1.** The scale used to assess participants’ affect. Ratings were collected using an online questionnaire administered via Google Forms.

**Breathing exercise**

“The recording will help you perform a breathing exercise. Remember to relax and breathe at a slow pace. A moving line will indicate when to inhale and when to exhale. Follow the line on the recording. You can also close your eyes and breathe according to the audio cue”.


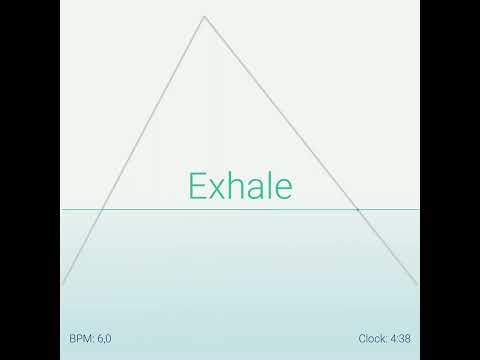


**Figure 2.** Screenshot from a video displaying a breathing pacer set to the participant’s prescribed breathing frequency. The screenshot was taken from the Paced Breathing application.

**Mood assessment after the breathing exercise**

“Please rate how you feel right now on a scale. Mark the dot that is closest to the adjective that describes how you feel at this moment”.

The subject rated their mood on the scale presented in Figure 1.
